# Garcinia Biflavonoid 1 Improves Lipid Metabolism in HepG2 Cells via Regulating PPARα

**DOI:** 10.3390/molecules27061978

**Published:** 2022-03-18

**Authors:** Hai-Xin Chen, Fan Yang, Xin-Qian He, Ting Li, Yong-Zhi Sun, Jian-Ping Song, Xin-An Huang, Wen-Feng Guo

**Affiliations:** 1 Artemisinin Research Center, Guangzhou University of Chinese Medicine, Guangzhou 510405, China; chenhaixin.chn@foxmail.com (H.-X.C.); flora.Yang0718@foxmail.com (F.Y.); charilce@foxmail.com (X.-Q.H.); tingli910@foxmail.com (T.L.); mugui0510@163.com (Y.-Z.S.); songjp@gzucm.edu.cn (J.-P.S.); 2 The First Affiliated Hospital, Guangzhou University of Chinese Medicine, Guangzhou 510405, China

**Keywords:** garcinia biflavonoid 1, PPARα, lipid metabolism, fatty acid oxidation

## Abstract

Garcinia biflavonoid 1 (GB1) is one of the active chemical components of *Garcinia kola* and is reported to be capable of reducing the intracellular lipid deposition, which is the most significant characteristic of non-alcoholic fatty liver disease. However, its bioactive mechanism remains elusive. In the current study, the lipid deposition was induced in HepG2 cells by exposure to oleic acid and palmitic acid (OA&PA), then the effect of GB1 on lipid metabolism and oxidative stress and the role of regulating PPARα in these cells was investigated. We found that GB1 could ameliorate the lipid deposition by reducing triglycerides (TGs) and upregulate the expression of PPARα and SIRT6, suppressing the cell apoptosis by reducing the oxidative stress and the inflammatory factors of ROS, IL10, and TNFα. The mechanism study showed that GB1 had bioactivity in a PPARα-dependent manner based on its failing to improve the lipid deposition and oxidative stress in PPARα-deficient cells. The result revealed that GB1 had significant bioactivity on improving the lipid metabolism, and its potential primary action mechanism suggested that GB1 could be a potential candidate for management of non-alcoholic fatty liver disease.

## 1. Introduction

Non-alcoholic fatty liver disease (NAFLD) is one of the most prevalent liver diseases, present in over 25% of the world population, and it is mainly identified by intracellular lipid deposition [1]. The “two-hit” modality has long been regarded as the prevailing notion behind the onset of NAFLD [2,3]. Recently, a new modality known as “multiple-hit” is emerging. It is proposed given the concurrent alterations of lipid oxidation and hyperactive proinflammatory factors owing to the numerous free fatty acids and reduced mitochondrial activity in liver cells that have been experiencing durable lipid deposition caused by dietary or genetic factors [4]. It might be effective to ameliorate the lipid deposition in liver cells in early NAFLD, conducive to the delay of progression of relevant phenotypes [5].

Peroxisome proliferator-activated receptor α (PPARα) is a meaningful transcription factor mainly restricted to the liver and playing a vital part in the process of lipid metabolism [6]. Increasing evidence has suggested that PPARα can be protective against NAFLD by regulating lipid metabolism, including facilitating the uptake and oxidation of fatty acids [7]. In addition, Sirtuin6 (SIRT6) is also a key regulator of lipid metabolism, and SIRT6 promotes liver beta-oxidation by activating PPARα [8]. It is identified that activation of PPARα can ameliorate the excess inflammation and cell apoptosis induced by intracellular lipid deposition. On the contrary, deficiency of PPARα can lead to hyperactivation of intracellular pro-inflammatory factors with the reduction of mitochondrial membrane potential (MMP), generating a large number of harmful substances, and subsequently resulting in persistent progression of NAFLD [9].

**Figure 1:**
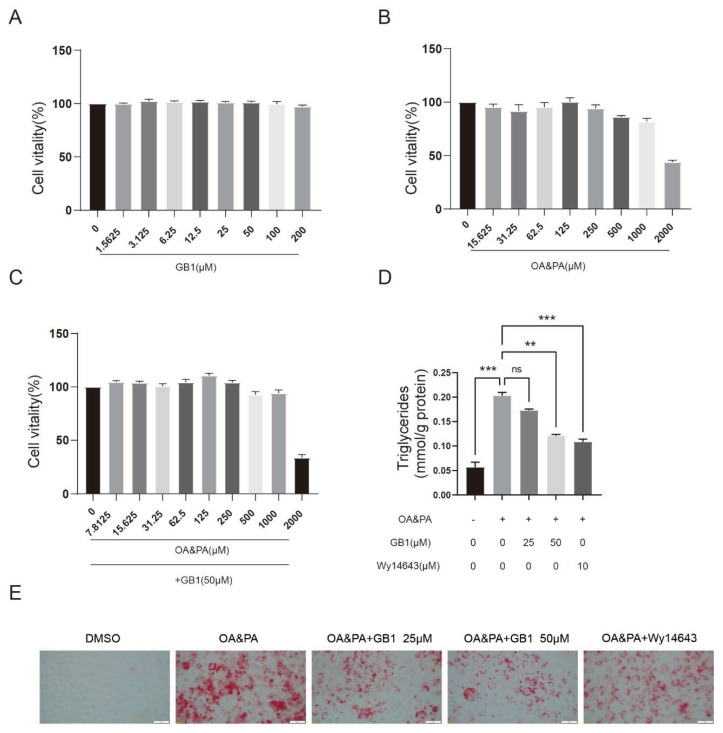

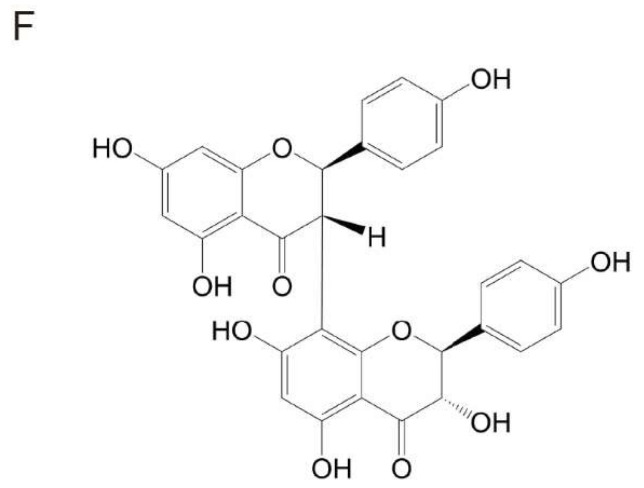
GB1 improves lipid deposition in liver cells. HepG2 cells were exposed to OA (500 µM) and PA (250 µM) for 24 h, and were then subjected to Wy14643 (PPARα agonist) and GB1 (25 µM, 50 µM) for 24 h. (**A**) HepG2 cell activity under different concentrations of GB1. (**B**) HepG2 cell activity under different concentrations of OA&PA. (**C**) HepG2 cell activity under 50 µM GB1 and different concentrations of OA&PA. (**D**) Triglycerides level in HepG2 cells under different conditions. (**E**) Representative Oil-Red-O stained images (400×). (**F**) Chemical structure of GB1. All data were presented as mean ± SEM of three independent experiments. **, *p* < 0.01, ***, *p* < 0.001; ns, not significant.

Garcinia biflavonoid 1 (GB1, chemical structure as showed in Figure 1F), the main active chemical composition of *Garcinia kola*, was reported to be capable of regulating glucose and lipid metabolism, inhibiting inflammation, and protecting the liver [10,11,12,13,14]. An animal experiment also identified the potential role of GB1 in the treatment of NAFLD, as it could decrease the blood glucose level induced by STZ in rabbits and was protective of the liver [15]. However, the specific molecular mechanism behind the action of GB1 for lipid metabolism in liver cells needs to be further clarified. We isolated compound GB1 from *Garcinia Kola Heckel* with a purity of 99.6% (Supplementary Material). The identification and analysis of the hydrogen (^1^H NMR) and carbon spectra (^13^C NMR) showed that the compound was GB1 (Supplementary Table S1 and Figure S1B,C). In the current study, lipid deposition was induced in HepG2 liver cells exposed to oleic acid and palmitic acid (OA&PA). In our GB1 studies on other diseases, we found that about 50 µM of GB1 had significant efficacy, so we referred to the effective concentrations of GB1 on other diseases [16]. GB1 was administrated to study its effect on intracellular lipid deposition, and the role of PPARα in that process was also explored.

## 2. Results

### 2.1. GB1 Effectively Ameliorates the Lipid Deposition in Cells

Lipid deposition in HepG2 cells was induced by exposure to OA&PA without too much cytoxicity (Figure 1B,C). Despite the low cytotoxicity (Figure 1A), GB1 significantly decreased the intracellular lipid deposition (Figure 1D). Oil-Red-O staining also demonstrated a decreased level of lipids in cells. These findings indicated that GB1 is effective in decreasing the lipid deposition in liver cells (Figure 1E).

### 2.2. GB1 Regulates Lipid Metabolism by Facilitating Lipid Oxidization and Decreasing the Level of Fatty Acids

**Figure 2:**
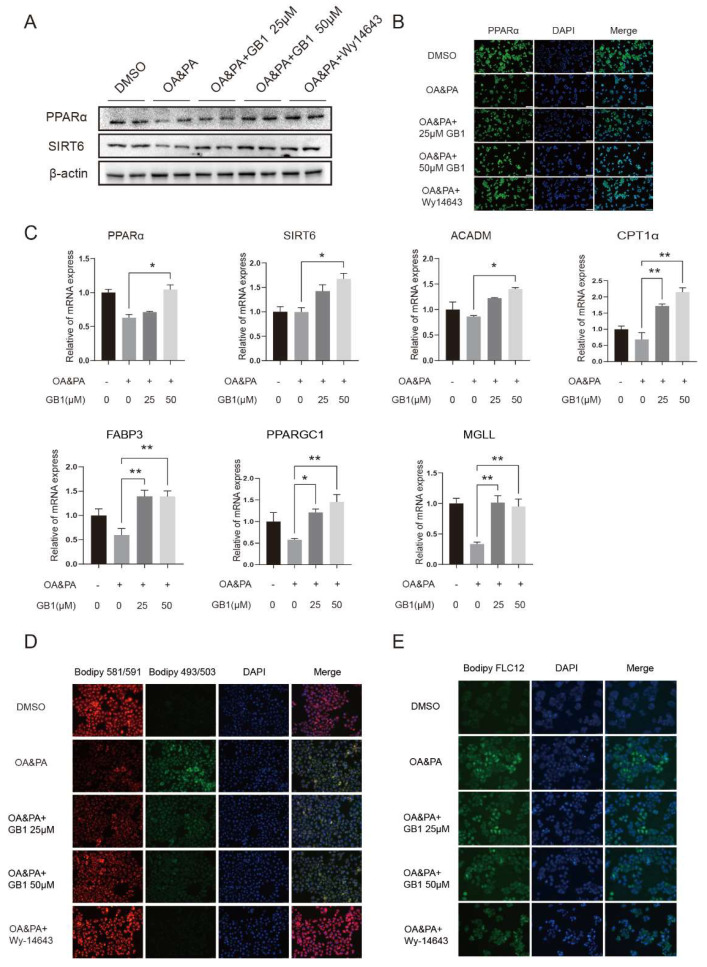
GB1 positively regulates lipid metabolism. (**A**) Western blot and (**B**) immunofluorescence staining assays revealed upregulation of the protein levels of PPARα and SIRT6 in HepG2 cells undergoing OA&PA culture after GB1 treatment. (**C**) Changes in mRNA levels of PPARα and SIRT6 after GB1 treatment. GB1 advanced lipid oxidization (**D**) and reduced the level of free fatty acids (**E**) in liver cells (200×). All data were presented as mean ± SEM of three independent experiments. *, *p* < 0.05, **, *p* < 0.01.

It was reported that intracellular lipid deposition is a significant characteristic in NAFLD. To explore the effect of GB1 on lipid metabolism, lipid oxidation genes PPARα and SIRT6 were measured. It was found that GB1 elevated the expression of PPARα and SIRT6 in both mRNA (Figure 2C) and protein levels (Figure 2A). In the meantime, other related genes also varied with differential degrees (Figure 2C). A consistent finding was obtained in immunofluorescence staining analysis for PPARα (Figure 2B). We also found advanced lipid oxidization and a reduced level of free fatty acids in cells after GB1 treatment (Figure 2D,E). Collectively, GB1 serves as an activator for lipid metabolism in liver cells.

### 2.3. GB1 Improves OA&PA Induced ROS Burden

**Figure 3:**
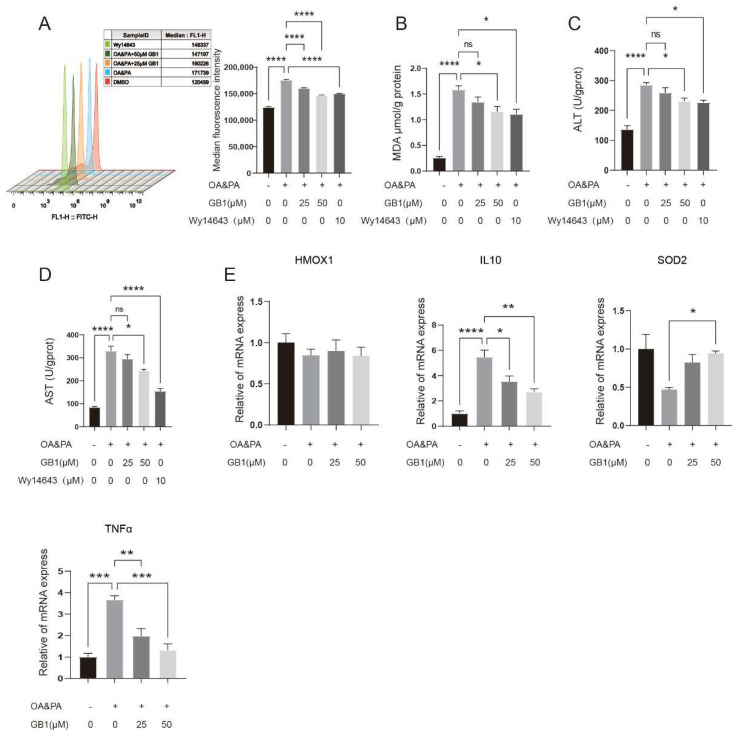
GB1 improves the ROS burden induced by OA&PA in HepG2 cells. (**A**) Flow cytometry showed the suppression of ROS level by GB1 treatment. (**B**) GB1 reduced the production of MDA, ALT (**C**) and AST (**D**). (**E**) GB1 suppressed apoptosis-related genes. All data were presented as mean ± SEM of three independent experiments. *, *p* < 0.05, **, *p* < 0.01, ***, *p* < 0.001, ****, *p* < 0.001; ns, not significant.

Increasing evidence has suggested that excess lipid deposition in cells can lead to overproduction of ROS, which severely damages cellular function and induces mitochondrial stress and subsequent persistent inflammatory responses. Here, suppression of the ROS overproduction induced by OA&PA was demonstrated after GB1 treatment (Figure 3A–D), with concurrently inhibited inflammatory genes, such as IL-10 and TNF-α (Figure 3E). Therefore, GB1 could reduce the level of inflammatory response induced by lipid deposition.

### 2.4. GB1 Reduces OA&PA Induced Apoptosis

**Figure 4:**
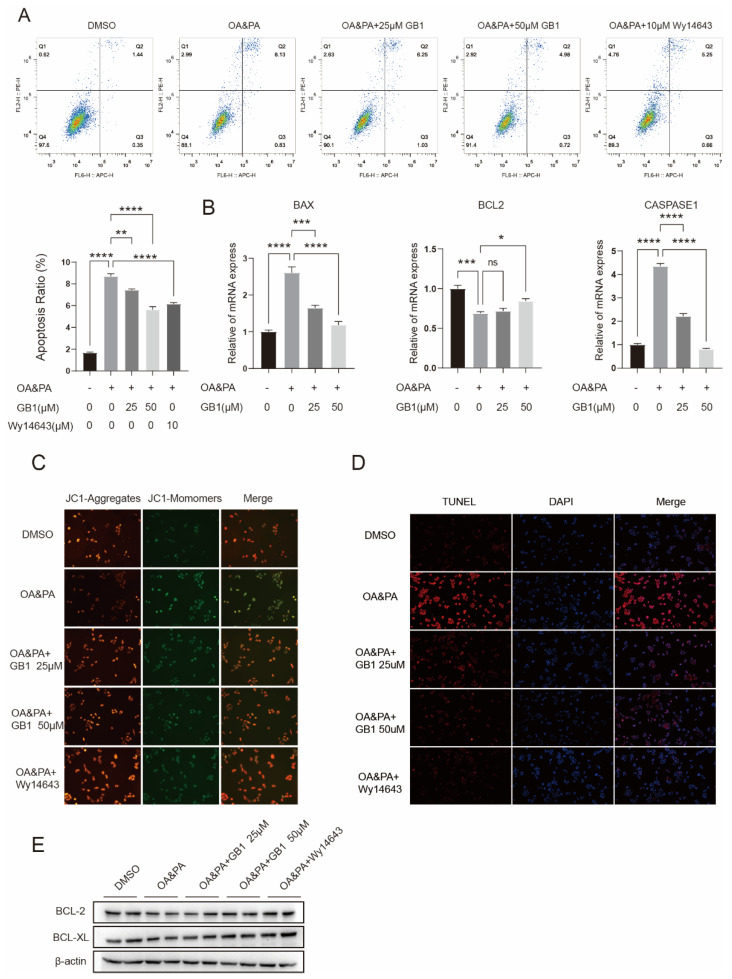
GB1 decreases the cell apoptosis induced by OA&PA. (**A**) Flow cytometry was performed to analyze cell apoptosis after treatment with GB1. (**B**) GB1 inhibited the expression of apoptosis-related genes. (**C**) Representative images for MMP after GB1 treatment (200×). (**D**) Representative images for TUNEL-labeled cells that underwent apoptosis (200×). (**E**) GB1 treatment elevated the expression of anti-apoptotic proteins. All data were presented as mean ± SEM of three independent experiments. *, *p* < 0.05, **, *p* < 0.01, ***, *p* < 0.001, ****, *p* < 0.001; ns, not significant.

Lipid deposition is known as one of the factors leading to cell apoptosis. As we had proven that GB1 could decrease the lipid deposition in cells, we then analyzed whether GB1 has certain effect on apoptosis. Expectedly, treatment with GB1 led to reduced cell apoptosis that had been induced by exposure to OA&PA (Figure 4A), and the genes associated with apoptosis were also inhibited (Figure 4B). In the meantime, the MMP was elevated (Figure 4C). TUNEL analysis also revealed a decreased number of cells undergoing apoptosis (Figure 4D). In addition, Western Blot showed that BCL-2 and BCL-XL expression was upregulated (Figure 4E). These findings demonstrated that GB1 is protective of cells against apoptosis induced by lipid deposition.

### 2.5. PPARα Is a Candidate Target of GB1

**Figure 5:**
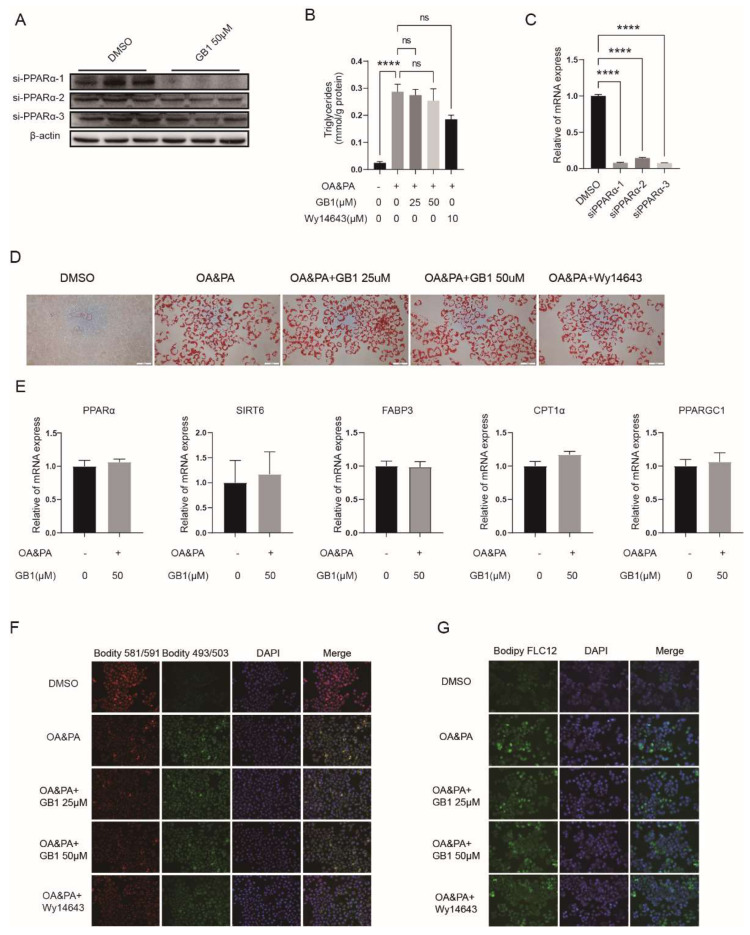
GB1 fails to improve the lipid deposition in PPARα-deficient cells. PPARα silenced by siRNA as demonstrated by Western blot (**A**) and RT-qPCR (**C**). (**B**) Level of triglycerides (TGs) in PPARα-deficient cells after treatment with GB1. (**D**) Representative image of lipid deposition in PPARα-deficient cells after treatment with GB1. (**E**) Levels of relevant genes in PPARα-deficient cells after treatment with GB1. No significant alterations in the levels of lipid oxidization (**F**) and free fatty acids (**G**) in PPARα-deficient cells after GB1 treatment under an inverted fluorescence microscope. All data were presented as mean ± SEM of three independent experiments. ****, *p* < 0.001; ns, not significant.

To explore the role of PPARα in the mechanism of action of GB1 for lipid deposition, PPARα was silenced in HepG2 cells cultured in OA&PA medium using targeting siRNA (Figure 5A,C). No alterations of lipid deposition in PPARα-silenced cells were noted after treatment with GB1 (Figure 5B). Consistently, no significant difference was observed in Oil-Red-O staining (Figure 5D) and expression of relevant genes (Figure 5E). Additionally, reduced pharmacological activity of GB1 was demonstrated for the levels of lipid oxidization (Figure 5F) and free fatty acids (Figure 5G) in PPARα-silenced cells.

### 2.6. GB1 Fails to Improve Inflammation and Apoptosis in PPARα-Deficient Cells

**Figure 6:**
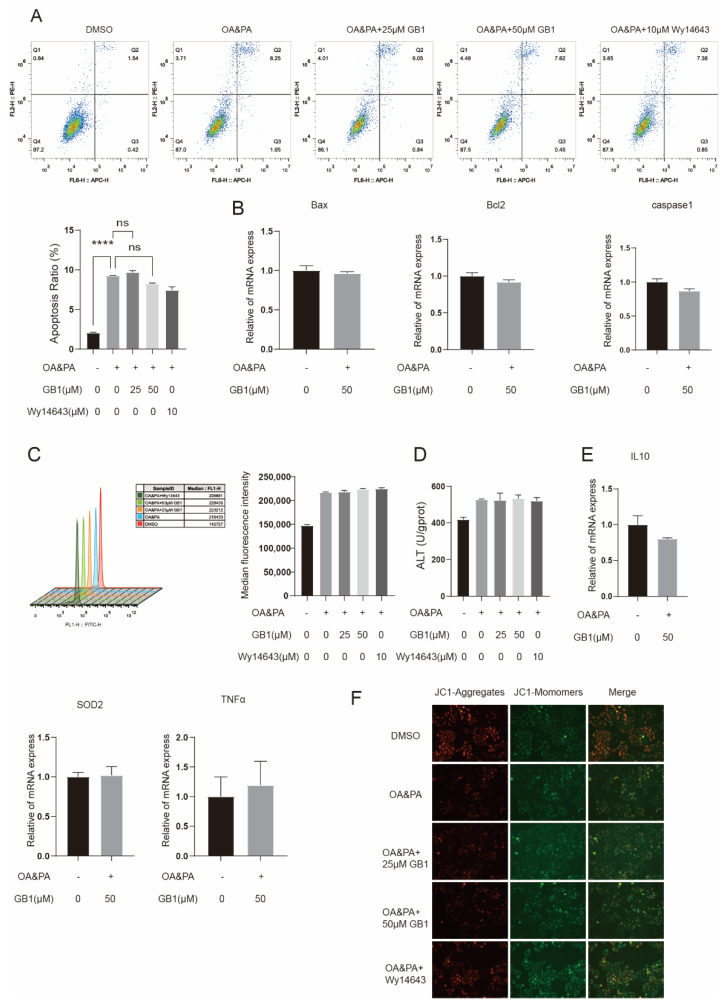
GB1 fails to improve inflammation and apoptosis in PPARα-deficient cells. ROS level (**A**) and apoptosis (**C**) in PPARα-deficient cells did not change after GB1 treatment. GB1 failed to alter the levels of inflammatory genes (**E**), apoptosis-related genes (**B**), ALT (**D**), and MMP (**F**) in PPARα-deficient cells. All data were presented as mean ± SEM of three independent experiments.****, *p* < 0.001; ns, not significant.

We found that there was no improvement in cell apoptosis in PPARα-silenced cells after treatment with GB1 (Figure 6A), and the expression of relevant genes was not altered (Figure 6B). Besides, no significant changes in MMP (Figure 6F), ROS level (Figure 6C), ALT (Figure 6D) and expression of inflammatory genes were examined (Figure 6E). In PPARα-silenced cells, GB1 tends to have poor pharmacological activity against the inflammatory response and apoptosis induced by lipid deposition.

## 3. Discussion

An intracellular lipid deposition is the most significant characteristic in NAFLD, and the secondary inflammation and cell apoptosis are risk factors of progression of this disease [17]. For the past few years, the incidence of NAFLD has been increasing in the world population, especially in developed countries [1]. In the current study, we noted that GB1 decreased the lipid deposition in cells exposed to OA&PA by inhibiting inflammation and enhancing the activity against lipid oxidation, which was in a PPARα-dependent manner.

Increasing evidence has suggested that NAFLD can be caused by multiple genetic or non-genetic factors [18]. A “multiple-hit” modality has been emerging in recent years and is being gradually accepted. It is established that lipid deposition in cells is a result of metabolic disturbance, which is largely due to the reduction in genes involved in lipid metabolism, such as PPARα and SIRT6 [19]. When lipid deposition occurs, the ROS will be largely secreted from cells, which changes the MMP and thereby damages the mitochondrial function to induce the production of numerous toxic substances including MDA and ALT [20]. In that way, there is a high risk of inflammation and apoptosis, ultimately resulting in the incidence of NAFLD [21]. Previous research demonstrated that GB1, a chemical component of Garcinia kola, is capable of improving the lipid metabolism in cells [22,23,24]. Here, we found that GB1 could effectively activate the expression of PPARα to ameliorate inflammatory responses and apoptosis in cells, which eventually decreased lipid deposition.

PPARα is a key regulator of lipid metabolism. There is abundant literature showing that the activation of PPARα could promote the intracellular lipid oxidation and reduce its deposition [25]. In addition, PPARα can reduce inflammation caused by lipid deposition in cells. PPARα agonists have a significant curative effect in reducing steatosis, inflammation, apoptosis, and the progression of tissue fibrosis in liver of mice on a high-lipid-induced diet. By contrast, in mice with liver-specific knockout of PPARα, mice could lead to non-alcoholic fatty liver even under normal conditions of diet [26]. In addition, SIRT6 is closely related to PPARα, and SIRT6 binds to the promoter region of PPARα and its reaction elements to activate gene transcription, thereby improving liver fat content [8]. Lipid metabolism abnormalities in nonalcoholic fatty liver can be defined as the changes in the content of intracellular TG. The results of Oil Red O staining and determination of TG content in our work proved that GB1 could alleviate intracellular lipid deposition. The outcome of intracellular fatty acid immunofluorescence was also consistent with the result above. Meanwhile, Western blotting and quantitative PCR showed that GB1 could promote the expression of PPARα. In addition, Western blotting showed that GB1 also up-regulated SIRT6 expression. PPARα may be a potential pharmacological target of GB1 thanks to its strong connection with lipid metabolism.

Under physiological conditions, liver function is strictly regulated to respond to environmental stress [27]. Excess fatty acids are a cause of intracellular lipid deposition, abnormal lipid peroxidation, release of pro-inflammatory factors, and accumulation of large amounts of ROS [28]. These substances lead to dysfunction of the endoplasmic reticulum and mitochondria, inducing oxidative stress and apoptosis. In our results, both Western blotting and RT-qPCR demonstrated that GB1 reduced apoptosis, while flow cytometry showed a decrease in intracellular ROS load. Immunofluorescence showed that GB1 alleviated intracellular lipid peroxidation and fatty acid reduction. Our results proved that GB1 alleviates intracellular inflammation and apoptosis caused by lipid deposition.

To further explore the critical role of PPARα in lipid metabolism, we silenced cellular PPARα with siRNA. The results of Western blot and RT-qPCR showed that siRNA successfully silenced PPARα. Interestingly, after PPARα was silenced by siRNA, the pharmacological activity of GB1 on HepG2 cells disappeared, and PPARα silencing eliminated the ameliorative effect of GB1 on lipid accumulation and oxidative stress in HepG2 cells. These suggest that PPARα may be a potential target of pharmacological activity.

## 4. Materials and Methods

### 4.1. Cell Activity

HepG2 cells were obtained from Shanghai Cell Bank of the China Academy of Sciences (Shanghai, China). HepG2 cells were inoculated in a 96-well plate at 5 × 10^3^ cells/well overnight. On the following day, the cells were exposed to a mixture of free fatty acids (OA/PA = 2:1) and then treated with GB1 for 24 h, compared with those not treated with GB1 as control. Cell activity was assessed with the CCK-8 assay kit.

### 4.2. Western Blot

HepG2 cells were firstly cultured with GB1 or Wy14643 (HY-16995, MedChemExpress, Monmouth Junction, NJ, USA) in a petri dish (6 cm). Following PBS washing two times, the cells were lysed in lysis buffer to obtain total proteins. Equal amounts of protein were separated by electrophoresis and then transferred to polyvinylidene fluoride (PVDF) membrane (Millipore, Burlington, MA, USA). The membrane was subsequently blocked with 5% skim milk, followed by incubation with anti-PPARα (ab61182, Abcam, Shanghai, China), anti-SIRT6 (A3591, ABclonal, Woburn, MA, USA), anti-BCL-2 (T40056, Abmart, Berkeley Heights, NJ, USA), anti-β-actin (RM3002, Beijing Ray antibody biotech, Beijing, China), and anti-BCL-XL (T55050, Abmart, Berkeley Heights, NJ, USA) antibodies. Relative expression of the proteins was normalized to actin protein. The total load of the protein was 60 µg/µL. The dilution ratio of primary antibody was 1:1000, and that of secondary antibody was 1:5000. The type of secondary antibody was Goat anti-Rabbit. Chemiluminescence signals were quantified using a chemiluminescence imaging system (BIO RAD, Hong Kong, China).

### 4.3. Real-Time Quantitative Polymerase Chain Reaction (RT-qPCR)

HepG2 cells were harvested to extract total RNA using Trizol reagent, following culture in a six-well plate and PBS washing two times. Reverse transcription was conducted using the RT-MIX to obtain cDNA. RT-qPCR amplification was performed with the SYBR GREEN MIX (4309155, Invitrogen, Waltham, MA, USA). RT-qPCR cycle conditions: 95 °C, 2 min; 95 °C, 15 s; 60 °C, 60 s; there were 40 cycles. Detailed primer sequences are listed in Table 1. RNA expression level was normalized to actin.

### 4.4. Immunofluorescence Staining

Here, 1 × 10^6^ HepG2 cells were cultured in a 12-well plate, fixed in 4% paraformaldehyde for 30 min, and then blocked in PBS + 1% BSA for 1 h at room temperature. Following that, the cells were incubated with primary antibodies (ab61182, Abcam, Shanghai, China) at 4 °C overnight. On the next day, the cells were incubated with secondary fluorescent-conjugated IgG (FITC-IgG) (AS011, ABclonal, Woburn, MA, USA) at room temperature. The primary antibody dilution concentration was 1:200 and the secondary antibody dilution concentration was 1:200. After 1 h, the cells were washed with PBS for three times. DAPI was used to counterstain the nuclei. An inverted fluorescence microscope was employed to capture images. All experiments were repeated three times.

### 4.5. Levels of Lipid Oxidization and Free Fatty Acids

Lipid oxidation in cells was examined using the fluorescent lipid probe (D3861, BODIPY™ 581/591 C11, Invitrogen, Waltham, MA, USA), and the level of fatty acids was assessed using the fluorescent fatty acid probe (BODIPY™ FLC12, Invitrogen, Waltham, MA, USA). According to the manufacturer’s instructions, the HepG2 cells were respectively incubated with the lipid and fatty acid probes (10 µ/mL) for 20 min in the dark. Subsequently, the cells were washed with buffer solution and then analyzed for levels of lipid oxidization and free fatty acids.

### 4.6. Flow Cytometry for Cell Apoptosis and ROS Generation

After routine culture in a six-well plate, the HepG2 cells were digested with EDTA-free trypsin, washed with PBS twice, and then harvested. For cell apoptosis, the cells were protected from light and stained with Annexin V-FITC (A35110, Invitrogen, Waltham, MA, USA) at room temperature. After 30 min, flow cytometry was performed to test cell apoptosis. For reactive oxygen species (ROS), the cells were harvested and incubated with ROS probe (CellROX^tm^, Invitrogen, Waltham, MA, USA) for 30 min. The level of ROS in cells was then measured using flow cytometry.

### 4.7. Mitochondrial Membrane Potential (MMP)

The MMP was measured by JC-1 dye using JC-1 (C006, Beyotime, Shanghai, China) Mitochondrial Membrane Potential Assay Kit. Briefly, cells were washed twice with PBS and then incubated at 4 °C in the dark. After 20 min, pre-cooled JC-1 working solution was applied to stain the cells twice. The MMP was observed under a fluorescence microscope.

### 4.8. TUNEL Staining

TUNEL (C1089, Beyotime, Shanghai, China) staining was performed using the TUNEL assay kit. Following the standard process, the cells undergoing apoptosis were labeled with fluorescein-dUTP and the green-stained cells were regarded as TUNEL-positive. Nuclei were visualized using DAPI.

### 4.9. Statistical Analysis

GraphPad Prism 9.0 was run to complete data analysis. All data were expressed as the mean ± standard error of the mean (SEM). One-way analysis of variance (ANOVA) with Tukey’s multiple comparison test was used for statistical analysis. A *p*-value no more than 0.05 was interpreted as statistically significant.

## 5. Conclusions

To conclude, this is the first study that reports the effective role of GB1 in ameliorating intracellular lipid deposition and improving the related inflammatory response and apoptosis, which might be dependent on the activation of PPARα expression. GB1 might be a potential therapeutic agent for treatment of NAFLD.

## Figures and Tables

**Table 1:** Primers used for real-time qRT-PCR.

Primer	Sequences
SIRT6	F:CCCACGGAGTCTGGACCAT
	R:CTCTGCCAGTTTGTCCCTG
PPARα	F:ATGGTGGACACGGAAAGCC
	R:CGATGGATTGCGAAATCTCTTGG
β-actin	F:CATGTACGTTGTATCCAGGC
	R:CTCCTTAATGTCACGCAGAT
ACADM	F:ACAGGGGTTCAGACTGTATT
	R:TCCTCCGTTGGTTATCCACAT
CPT1α	F:TCCAGTTGGCTTATCGTGGTG
	R:TCCAGAGTCCGATTGATTGC
FABP3	F:GGCACCTGGAAGCTAGTGG
	R:CTGCCTGGTAGCAAAACCC
PPARGC1	F:TCTGATCTTATGGATGACAT
	R:CCAAGTCGTTCACATCTATTA
MGLL	F:ATGCCAGAGGAAAGTTCCCC
	R:CGTCTGCATTGACCAGGTG
HMOX1	F:AGACTGCGTTCCTGCTCAAC
	R:AAGCCCTACACAACTGTCG
IL10	F:GACTTTAAGGGTTACTGGGTTG
	R:TCACATGCGCCTGATGTCTG
Bax	F:CCCGGAGGTCTTTTTCCGAG
	R:CCAGCCCATGATGGTTCTGAT
CASPASE 1	F:TTTCCGCAAGGTTCGATTTCA
	R:GGCATCTGCGCTCTACCATC
BCL-2	F:GGTGGGGTCATGTGTGTGG
	R:CGGTTCAGGTACTCATCATCC

## Data Availability

All data generated or analyzed during this study are included in this article.

## Supplementary Materials

The following are available online at https://www.mdpi.com/article/10.3390/molecules27061978/s1, Figure S1: (A) GB1with the purity of 99.6%; (B) ^1^H-NMR spectrum of GB1; (C) ^13^C-NMR spectrum of GB1; Table S1: The NMR spectral data of GB1 (DMSO-*d*6).

## Sample Availability

Samples of the compounds garcinia biflavonoid 1 are available from the authors.
