# Supplementary material for: Garcinia Biflavonoid 1 Improves Lipid Metabolism in HepG2 Cells via Regulating PPARα
Source: Molecules. 2022 Mar 18;27(6):1978. doi: 10.3390/molecules27061978 (PMC8950208; doi:10.3390/molecules27061978)
Supplement: Supplementary file 1 [file molecules-27-01978-s001.zip › molecules-1611791-supplementary.pdf]

Methods

Transfection of small interfering RNA (siRNA).

At logarithmic growth stage, 3×10^6 cells were incubated on 6-well plates, and transfection began when cell density reached 30%. Diluted 1.25 μL 20 μM siRNA storage solution (v3) with 30 μL 1X ribo*FECT*TMCP Buffer (v2) and mixed gently. Add 3 μL ribo*FECT*TMCP Reagent (V4), blew gently, mixed, and incubated at room temperature for 15 min to prepare the transfection compound. The ribo*FECT*TMCP transfection complex was added into the proper amount of complete medium without double resistance (v1) and mixed gently. 50 μM GB1 cells were added and cultured for 48 h.

Extract Preparation.

Garcinia kola nuts were obtained from Nigeria, Africa. *Garcinia kola* nuts were cleaned using fresh tap water to remove dust, air dried, and then crushed. Extraction was performed twice using 95% (v/v) ethanol and then the solution was evaporated to semi dryness using a rotary vacuum evaporator at 60 ℃. The filter residue was added to pure water and refluxed for extraction twice for 1 h. The filtrate was combined and concentrated under reduced pressure to obtain the extract. All of the obtained extracts were dissolved in an appropriate amount of water for extraction with petroleum ether reagent. The water layer was further extracted three times with *n*-butanol. The *n*-butanol layer was concentrated under reduced pressure for subsequent purification.

HPLC parameters: extract powder was dissolved in methanol and filtered through a 0.22 μm filter for before High-Performance Liquid Chromatography (HPLC, Agilent Technologies, Santa, Clara, CA, USA) analysis with a Zorbax Eclipse Plus C_{18} column (ZORBAX SB-C_{18}, 9.4 × 250 mm, 5 μm, Agilent, USA). Chromatographic separation was performed at 30 ℃ with a flow rate of 2.5 mL/min. The ultraviolet detection wavelength was set at 280 nm. The mobile phase consisted of methanol (A) and water (B). The gradient elution conditions of the mobile phase A were: 0–10 min, 40%; 11–17 min, 32%; 18–21 min, 45%. After purification by HPLC, GB1 with the purity of about 99.6% was obtained.

Cell Protocols.

HepG2 cells were obtained from Shanghai Cell Bank of the China Academy of Sciences (Shanghai, China). HepG2 cells were cultured in DMEM high glucose medium (C11995500BT, gibco, USA) with 10% FBS (FSD500, ExcellBio, Australia), 1% penicillin and streptomycin (15140122, gibco, USA) in an incubator at 37 °C and 5% CO_2 concentration. GB1 was dissolved with DMSO (ST038, Beyotime, China) as a carrier and further diluted. After cell apposition, the cells were cultured in medium with 500 uM OA and 250 uM PA for 24 h. Then 25 μM and 50 μM GB1 were added to the cells. The same volume of DMSO was added to the control cells. To validate this model, Wy14643 (10 μM), a PPARα agonist, was used as a positive control. The cells were cultured for 24 h. The final concentration of DMSO in the cell culture medium was maintained at 0.1% (v/v) throughout the study. All experiments were repeated three times.

Preparation of OA&PA.

0.04 g NaOH (G19852H, Greagen) was dissolved in 10 mL distilled water. Preparation of oleic acid mother liquor: 0.06 g fatty acid-free BSA (A8850, SolarBio,

China) was dissolved in PBS (C11995500BT, Gibco, USA) at 55 ℃ and centrifuged at 8000 r/min to obtain 3 mL 20% fatty acid-free BSA solution. 19.04 uL OA (815202, Macklin, USA) was added to 3 mL NaOH solution and fully saponified in 75 ℃ water bath for about 30 min to obtain 2 mM sodium oleate saponified solution. Add the insulated OA solution to BSA solution quickly to get 6 mL 10 mM OA solution. Preparation of palmitic acid mother solution: 1.2 g of fatty acid-free BSA was dissolved in PBS at 55 ℃ and centrifuged at 8000 r/min to obtain 3 mL of 40% fatty acid-free BSA solution. 0.0307 g PA (H8780, Solarbio, China) was added into 3 mL NaOH solution and placed in 75 ℃ water bath for full saponification for about 30 min to obtain 40 mM sodium palmitate saponification solution. The insulated PA solution was quickly added to the BSA solution to obtain 6 mL 20 mM PA solution. Finally, the mother liquor of oleic acid and palmitic acid are mixed and diluted to the target concentration.

Cytotoxicity assay.

The cytotoxicity of GB1 and/or OA&PA was determined by CCK-8 assay. The cells were cultured in 96-well plates at a density of 5×10^4 and treated with different concentrations of GB1 (200, 100, 50, 25, 12.5, 6.25, 3.125, 1.5625, 0 μM) and/or OA&PA (2000, 1000, 500, 250, 125, 62.5, 31.25, 15.625, 0 μM). After 24 h culture, 10 μL of CCK-8 solution was added and cultured at 37 °C for 2 h. Absorbance was measured at 450 nm with a microplate reader. Cell viability was calculated according to the manufacturer's instructions.

Analysis of cellular lipids.

Triglycerides concentrations were determined by kit (Jiancheng, Nanjing, China). Cells were washed twice with PBS, lysed by lysis solution according to the manufacturer's instructions and the supernatant was collected. Blank wells, calibration wells and sample wells were set up and absorbance was measured at 510 nm with a microplate reader. Total TGs levels were normalized to g of protein as determined by the BCA assay.

Supplementary Table

Table S1. The NMR spectral data of GB1 (DMSO-*d6*)

NO	GB1	
	δ H	δ C
I-C-3	4.65 (*d*, 12.1)	47.49, 47.39
	4.40 (*d*, 12.1)	
II-C-3	4.21(*d*, 11.2)	72.47, 71.98
	3.99 (*d*, 11.2)	
I-C-2	5.65 (*d*, 12.1)	81.80, 81.26
	5.30 (*d*, 12.1)	
II-C-2	5.12 (*d*, 11.2)	82.59, 82.54
	4.95 (*d*, 11.6)	
I-A-6	5.93 (*s*, 1H)	96.18, 95.97
	5.80 (*s*, 1H)	95.45
I-A-8		

II-A-6	5.76 (*s*, 1H)	95.10, 95.02
II-A-8		101.34
I-A-4a		101.38, 101.44
II-A-4a		99.70, 100.24
I-B-3'	6.74 (*d*, 8.2)<br>6.64 (*d*, 5.9)	114.69, 114.71
I-B-5'	6.74 (*d*, 8.2)<br>6.64 (*d*, 5.9)	114.81, 115.01
II-B-2'	7.16 (*d*, 4.5)	128.24
II-B-5'	6.83 (*d*, 8.2)<br>6.64 (*d*, 5.9)	114.81, 115.01
II-B-6'	7.16 (*d*, 4.5)	128.24
I-B-1'		129.16, 128.83
Il-B-1'		129.05, 128.83
I-B-2'	7.09 (*dd*, 14.8,8.3)	128.01, 127.92
1-B-6'	7.09 (*dd*, 14.8,8.3)<br>8.3	127.66
II-B-3'	6.83 (*d*,8.2)<br>6.64 (*d*,5.9)	114.81, 115.01
II-B-4'	9.57 (*s*)<br>9.45 (*s*)	157.89,157.81
I-B-4'	9.57 (*s*)<br>9.45 (*s*)	157.63, 157.41
I-A-5	12.29 (*s*)<br>12.17 (*s*)	163.73, 163.93
I-A-7	10.78	166.40, 166.53
I-A-8a		162.70, 162.83
II-A-5	11.86 (*s*)<br>11.73 (*s*)	161.90, 162.33
II-A-7	10.78	165.03, 165.33
II-A-8a		159.46, 160.24
I-C-4		196.54, 196.89
II-C-4		197.47
II-C-3-OH	5.95	

A: spectra data; B: documentation data (DOI: 10.1055/s-2006-962704)

Supplementary Figures

Figure legends

Figure S1.

(A) GB1with the purity of 99.6%.

(B) ^{1}H-NMR spectrum of GB1

(C) ^{13}C-NMR spectrum of GB1

Supplementary Figure S1

A

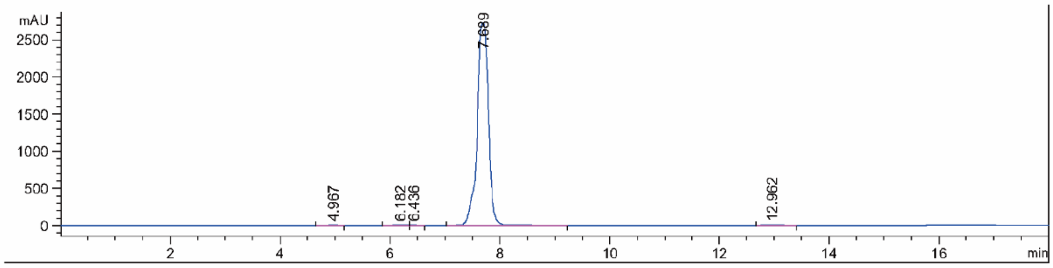
mAU
2500
2000
1500
1000
500
0
4.967
6.182
6.436
7.689
12.962
2
4
6
8
10
12
14
16
min

B

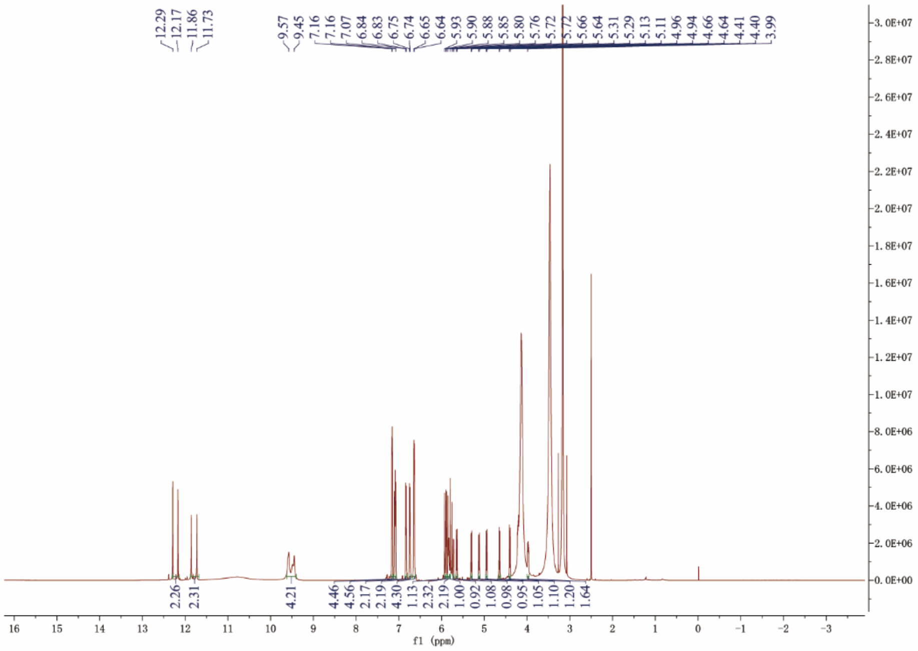
12.29 12.17 11.86 11.73
9.57 9.45 7.16 7.16 7.07 6.84 6.83 6.75 6.74 6.65 6.64 5.93 5.90 5.88 5.85 5.80 5.76 5.72 5.72 5.66 5.64 5.31 5.29 5.13 5.11 4.96 4.94 4.66 4.64 4.41 4.40 3.99
2.26 2.31 4.21 4.46 4.56 2.17 2.19 4.30 1.13 2.32 2.19 1.00 0.92 1.08 0.98 0.95 1.05 1.10 1.20 1.64
f1 (ppm)

C

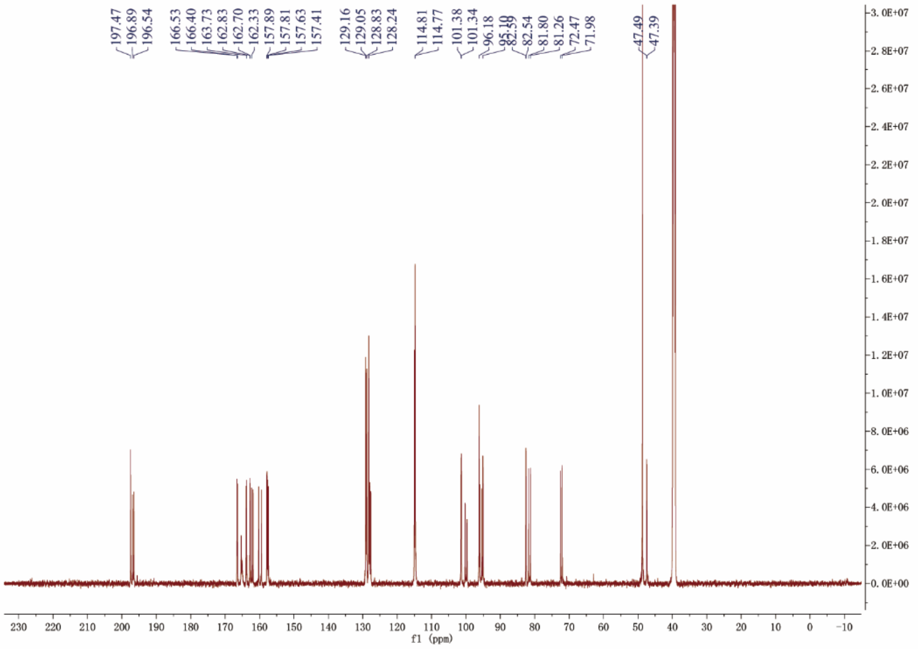
197.47 196.89 196.54
166.53 166.40 163.73 162.83 162.70 162.33 157.89 157.81 157.63 157.41
129.16 129.05 128.83 128.24
114.81 114.77
101.38 101.34 96.18 95.10 82.59 82.54 81.80 81.26 72.47 71.98
47.49 47.39
f1 (ppm)
